# Role of Recruitment Processes in Structuring Coralligenous Benthic Assemblages in the Northern Adriatic Continental Shelf

**DOI:** 10.1371/journal.pone.0163494

**Published:** 2016-10-04

**Authors:** Federica Fava, Massimo Ponti, Marco Abbiati

**Affiliations:** 1 Dipartimento di Scienze Biologiche, Geologiche ed Ambientali (BiGeA) & Centro Interdipartimentale di Ricerca per le Scienze Ambientali (CIRSA), University of Bologna, CoNISMa, Ravenna, Italy; 2 Istituto di Scienze Marine, Consiglio Nazionale delle Ricerche, Bologna, Italy; Universita degli Studi di Genova, ITALY

## Abstract

Coralligenous biogenic reefs are among the most diverse marine habitats in the Mediterranean Sea. The northern Adriatic mesophotic coralligenous outcrops host very rich and diverse epibenthic assemblages. Several studies quantified the low temporal variability and high spatial heterogeneity of these habitats, while processes driving structuring and differentiation are still poorly understood. To shed light on these processes, temporal and spatial patterns of colonisation were investigated using travertine tiles deployed on three coralligenous outcrops, corresponding to the main typologies of benthic assemblages described in previous studies. Three years after deployment, assemblages colonising travertine tiles resembled the differentiation among sites revealed by the natural assemblages in terms of major ecological groups. Processes structuring and maintaining species diversity have been explored. Pioneer species with high reproduction rate, long distance larval dispersal and fast growth (e.g. the serpulid polychaete *Spirobranchus triqueter* and the bivalve *Anomia ephippium*), were the most abundant in the early stages of recruitment on the two outcrops further away from the coast and with lower sedimentation. Their success may vary according to larval availability and environmental conditions (e.g., sedimentation rates). At these sites early-stage lasted 10–12 months, during which even species from natural substrates began colonising tiles by settlement of planktonic propagules (e.g., encrusting calcareous Rhodophyta) and lateral encroachment (e.g., sponges and ascidians). On coastal outcrop, exposed to a higher sedimentation rates, tiles were colonised by fast-growing algal turfs. Resilience of northern Adriatic coralligenous assemblages, and maintenance of their diversity, appeared largely entrusted to asexual reproduction. Exploring the mechanisms that underlie the formation and maintenance of the species diversity is crucial to improve our understanding of ecological processes and to implement appropriate conservation strategies of the Adriatic coralligenous reefs.

## Introduction

The Mediterranean coralligenous habitats are mesophotic biogenic temperate reefs formed mainly by the accumulation of calcareous encrusting algae growing in dim light conditions between 20 and 120 m depth [1]. The depth range of these reefs depends primarily on solar irradiance attenuation, which in turn is affected by water turbidity (i.e. suspended particles and plankton [2]). Structures of the assemblages are shaped by the growth patterns of the dominant algal species, together with lithification and biological and physical erosion processes. These habitats host the highest level of benthic species diversity in the Mediterranean Sea [3]. Given their ecological and economic importance, Mediterranean coralligenous habitats are included among the natural habitats of European Community interest, deserving the designation of special areas of conservation (European Habitat Directive, 92/43/EEC; [4]). They are also listed in the Barcelona Convention for protection against pollution in the Mediterranean Sea, in the framework of the Regional Seas Programme of the United Nations Environment Programme [5].

Two major forms of coralligenous habitats have been described: rims, growing on coastal rocks (e.g., vertical cliffs, overhangs and outer part of marine caves); platform outcrops, developing on the continental shelves over consolidated sediments, coalesced rhodoliths or pre-existing rocky substrates [1]. Since the ‘60s a bulk of data on species dwelling on Mediterranean biogenic reefs has been collected, suggesting that these reefs encompass a number of different biogenic formations [6] and highlighting their complexity and diversity. Quantitative studies revealed the complexity in pattern of spatial variation, with most of the variation occurring at the local scales, while patterns of species composition at the regional scale were more consistent [7–13].

Most studies on spatial and temporal variability of coralligenous assemblages focused on coastal rocky cliffs, especially on the macroalgal component [14–17], while assemblages on platform outcrops were largely unexplored (but see [18]).

Sandy and muddy bottoms in the northern Adriatic continental shelf host a large number of coralligenous subtidal reefs. They range in size from few to thousands square metres, and are emerging for up to 4 metres from the sedimentary bottoms. Distance between outcrops may range from few meters to tens of kilometres. Diversity of assemblages of northern Adriatic coralligenous outcrops has been documented [19–24]. Outcrops differ in species composition, revealing a high spatial heterogeneity, while temporal variability is mainly related to inter-annual fluctuations in abundances of taxa, without clear patterns [21]. According to Ponti et al. [21], off Chioggia and Venice three main typologies of benthic assemblages have been identified on the basis of the abundance of encrusting calcareous Rhodophyta, algal turfs, sponges and ascidians. Outcrops close to the shore were dominated by algal turfs and encrusting sponges. Small outcrops furthest from the coast were characterised by red calcareous algae and colonial ascidians. On outcrops in between, both algal turf and encrusting algae were abundant. Evidences that epibenthic assemblages on northern Adriatic coralligenous reefs differed according to their distance from the coast, depth, water turbidity and sediment load were already provided by Ponti et al. [21] and Curiel et al. [22]. These data were used to develop a predictive habitat distribution model intended to map areas having suitable environmental condition to host outcrops with different typologies of benthic assemblages [24]. Nevertheless, the ecological processes that lead to the formation of the assemblages and to the maintenance of their diversity are almost unknown.

Settlement and recruitment processes strongly influence the composition of benthic assemblages [25,26]. In turn, local variation in recruitment could be affected by micro-scale habitat heterogeneity [27,28], biotic interactions [29–31], larval supply [32] and larval behaviour [33].

The role of the colonisation processes in the formation and maintenance of the heterogeneity of the coralligenous assemblages on the northern Adriatic shelf has been investigated in a three-year recruitment experiment by deploying travertine tiles in the three main types of assemblages. Temporal patterns of assemblage formation were analysed. After three years, the assemblages developed on tiles were compared to those on neighbouring natural substrates to measure in which extent they may reflect the natural spatial heterogeneity.

## Materials and Methods

### Experimental setup and sample analysis

**Fig 1:**
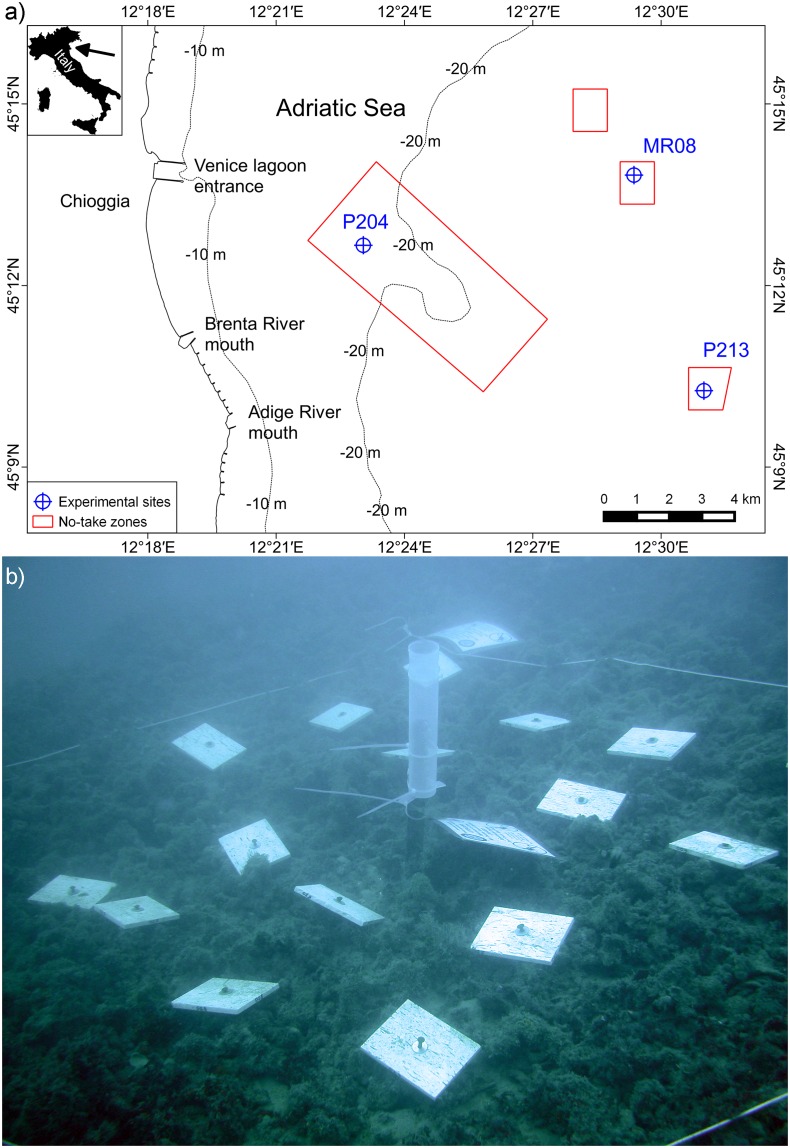
Sites location and experimental setup. (a) sketch map of the study area, showing the three experimental sites and the no take zone (NTZ) limits (for illustrative purposes only); (b) example of plot of travertine tiles and sediment trap, just deployed in P204 in August 2005.

One study site for each of the three main typologies of benthic assemblages recognised by Ponti et al. [21] was randomly selected (sites were labelled MR08, P204 and P213 as in previous publications, and all of them are included in the No-Take Zone established in 2002; Fig 1a). Selected sites were located between 6.6 and 15.0 km from the coast at 20.2–25.4 m depth. The closest to the coast (P204) was a large outcrop while the other two were small rocky areas (MR08, P213; Table 1). Three experimental plots, tens metres apart, were randomly selected within each site, and 16 rough travertine tiles (15.0 x 11.5 x 1.0 cm) were deployed in each plot in August 2005 (Fig 1b). Travertine is a form of porous limestone made by a process of rapid precipitation of calcium carbonate in hot springs or limestone caves. The material of the tiles was chosen as similar as possible to the natural biogenic substrate. Moreover, travertine is suitable for colonisation of boring species [34]. Compared to compact limestone, employed travertine had a mean porosity of 7%. Each tile was anchored to the bottom by means of steel nails and positioned horizontally to avoid any effect due to the substrate orientations [35]. Tiles were photographically sampled in January, June, August and October 2006, August 2007 and August 2008. At the end of the experiment (August 2008) assemblages on natural substrate close to each experimental plot were photographically sampled. Sampling was done using an Olympus Cammedia C-7070 WZ underwater digital camera (image size 3072 × 2304 pixels) equipped with a TTL strobe and two 50 Watt halogen lights. Photo-samples on natural assemblages were carried out using a steel frame with the same area as the experimental tiles.

**Table 1:** Location and geomorphological features of the of studied coralligenous outcrops.

Site	Latitude N	Longitude E	Depth	Coast Distance	Extent	Height
	(datum WGS84)		(m)	(km)	(m^2^)	(m)
**MR08**	45° 13.831'	12° 29.354'	22.2	14.6	2,096	1.5
**P204**	45° 12.674'	12° 23.076'	20.2	6.6	276,297	2.2
**P213**	45° 10.270'	12° 31.013'	25.4	15.0	1,288	1.2

Four randomly selected photo-samples, of the 16 available for each experimental plot and sampling date were analysed. The poor visibility prevented the finding of one plot at MR08 site in June 2006 and of two plots at P213 site in October 2006. Moreover one experimental plot at P213 site was lost since August 2007, maybe removed by fishing gears used in this not patrolled area of the No-Take Zone.

Percent cover of sessile organisms was quantified by superimposing a grid of 100 equal-sized squares and by identifying all taxa visible within each quarter of these squares, i.e. ±0.25%. Organisms were identified to the lowest possible taxonomic level. The endolithic bivalve *Rocellaria dubia* (Pennant, 1777) was quantified by counting its typical ‘8-shaped’ aragonitic siphonal tubes. Abundances of all taxa were adjusted to the total readable area of each image, obtained by subtracting dark and blurred zones or portion covered by motile organisms as in Ponti et al. [21].

Mean annual sedimentation rates were estimated at each plot using sediment traps (mouth diameter 55 mm, volume 500 ml, Fig 1b). Traps were replaced and the accumulated sediment measured whenever possible.

### Statistical analysis

Mean similarities between sessile assemblages on tiles, at each date and site, and those found on the corresponding local natural substrates in August 2008, three years after the beginning of the experiment, were measured by the Bray-Curtis index calculated on square root-transformed data. Differences in species percent covers and assemblage structures between Substrates (2 levels, fixed: travertine tiles and natural substrates), among Sites (3 levels, fixed: P204, MR08, P213) and Plots (3 levels, random, nested in Sites × Substrates) in August 2008 were tested by three-way permutational analysis of variance (PERMANOVA; [36,37]).

Colonisation patterns on tiles were analysed in term of assemblage structure, single species percent cover and species richness (*S*). Differences among Dates (6 levels, fixed: January, June, August and October 2006, August 2007, August 2008), Sites (3 levels, fixed: P204, MR08, P213) and Plots (3 levels, random, nested in Sites) were analysed by three-way PERMANOVA. Univariate tests were based on the Euclidean distances calculated on untransformed data [37], while multivariate tests were based on Bray-Curtis similarities on square root-transformed data [38]. Significant results were further analysed by ‘*a posteriori*’ pair-wise tests. When less than 999 unique values in the permutation distribution were available, asymptotical Monte Carlo *p*-values (*p*_*MC*_) were used instead of permutational *p*-values. Similarity patterns among assemblages were graphically represented by unconstrained ordination plots obtained using the principal coordinate analysis (PCoA, i.e. metric multidimensional scaling; [39]). Statistical analysis was performed using PRIMER 6 with PERMANOVA+ add-on package [40]. Mean values were reported along with their standard errors (± SE).

## Results

### Comparing assemblages on natural substrates and travertine tiles

**Fig 2:**
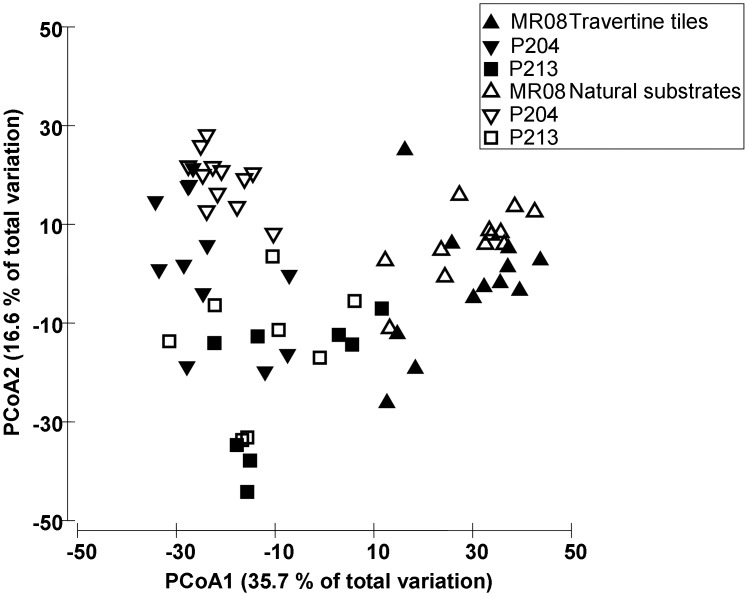
Similarities between assemblages on experimental tiles and on natural substrates three years after tiles deployment. PCoA ordination plot based on Bray-Curtis similarities of square root-transformed benthic assemblages data: each point was the centroid of the observed similarity among Sites and Substrates.

Three years after tiles deployment (August 2008), assemblages on travertine tiles and on natural substrates were still different. Overall, assemblages significantly differed among sites, but differences between natural substrate and artificial tiles were consistent across sites (Table 2). The PCoA plot, which represented 52.3% of the total variation, showed a clear separation among assemblages at different sites, and the shifts between the two substrates, within site, were similar at all sites (Fig 2).

**Table 2:** PERMANOVA test on Bray-Curtis similarities of square root-transformed benthic data according to Site (3 levels, fixed: MR08, P204, P213), Substrate (2 levels, fixed: travertine tiles and natural substrates) and Plot (3 levels, random, nested in Site × Substrate).

Source	df	SS	MS	Pseudo-*F*	*p* (perm)	Unique perms
Site	2	44189	22094.0	13.2370	0.0001	9937
Substrate	1	9899	9898.6	5.9303	0.0010	9939
Site × Substrate	2	4027	2013.5	1.2063	0.2755	9918
Plot (Site × Substrate)	10	16692	1669.2	2.2650	0.0001	9829
Res	48	35373	736.9			
Total	63	110320				

**Fig 3:**
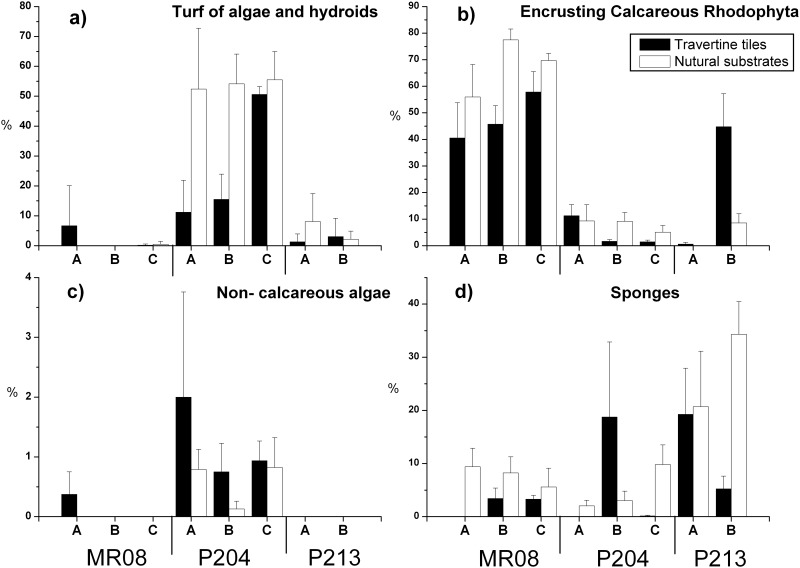
Abundance of most abundant taxa on experimental tiles and natural substrates three years after tiles deployment. Mean (± SE) percent cover of (a) turf of algae and hydroids, (b) encrusting calcareous Rhodophyta, c) non-calcareous algae and d) sponges, at each plot and site, both on travertine tiles and natural substrates, at the end of the recruitment experiment (August, 2008).

At the end of the recruitment experiment, assemblages were composed by four main ecological groups: mixed turf of filamentous algae and hydroids, encrusting calcareous Rhodophyta, non-calcareous algae and sponges. Abundance of these ecological groups significantly differed between sites but not between artificial and natural substrates (Table 3, Fig 3). Therefore, although assemblages on experimental tiles differed from the natural assemblages in species composition, they did not in terms of ecological groups.

**Table 3:** Summary of PERMANOVA test on percent cover of the main ecological groups at different Sites in relation to Substrates and among experimental Plots.

	Site			Substrate			Site × Substrate			Plot (Site × Substrate)			Res
	MS	*F*_*2*,*10*_	*p*	MS	*F*_*1*,*10*_	*p*	MS	*F*_*2*,*10*_	*p*	MS	*F*_*10*,*48*_	*p*	MS
Turf of algae and hydroids	10681.00	27.03	0.0004 ***	1445.70	3.66	0.0829 ns	1537.60	3.89	0.055 ns	395.13	5.66	0.0001 ***	69.82
Encrusting calcareous Rhodophyta	18017.00	30.44	0.0003 ***	31.94	0.05	0.8229 ns	1743.20	2.94	0.0922 ns	591.94	3.40	0.0028 **	174.00
Non-calcareous algae	5.66	10.82	0.0054 **	1.03	1.96	0.2014 ns	0.64	1.21	0.3606 ns	0.52	0.53	0.9328 ns	0.99
Sponges	1275.70	6.72	0.0202 *	647.60	3.41	0.0964 ns	330.35	1.74	0.2304 ns	189.85	1.57	0.1289 ns	120.82

Significant levels were indicated by the following symbols: ns = not significant,

* = *p* < 0.05;

** *p* < 0.01;

*** *p* < 0.001

**Fig 4:**
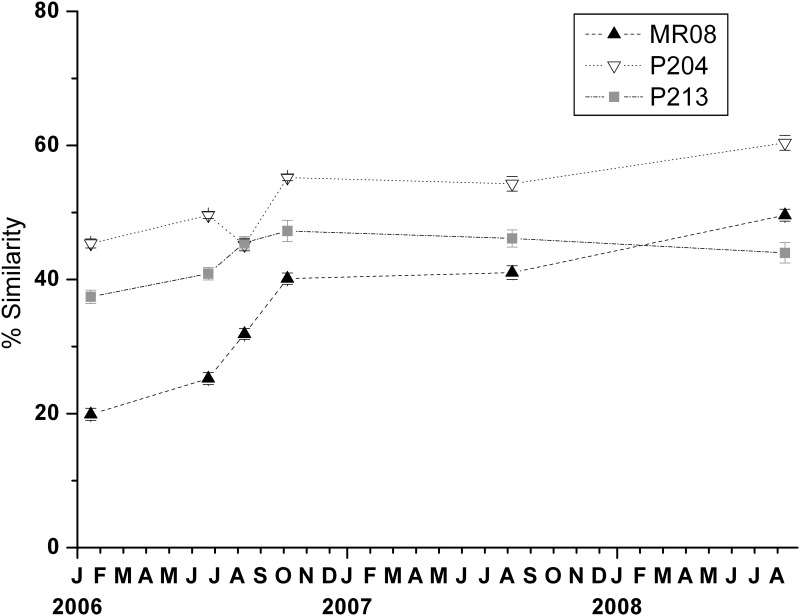
Temporal trends of similarity between the assemblages on experimental tiles and natural substrates at the end of the experiment. At each site and date mean similarity (±SE) of assemblages colonising tiles with the respective assemblages on natural substrates in August 2008 was calculated.

Overall, over the three-year period of the experiment, an increase in similarity between the benthic assemblages on tiles and on the nearby natural substrates, sampled in August 2008, was found (Fig 4). In particular, the mean similarity values increased from 19.89% to 49.6%, and from 45.4% to 60.4% at MR08 and P204, respectively. Similarity between natural and tiles assemblages at P204 appeared unexpectedly high since the beginning; while at P213 it gradually increased until October 2006 then slightly decreased.

### Recruitment dynamics on travertine tiles

**Fig 5:**
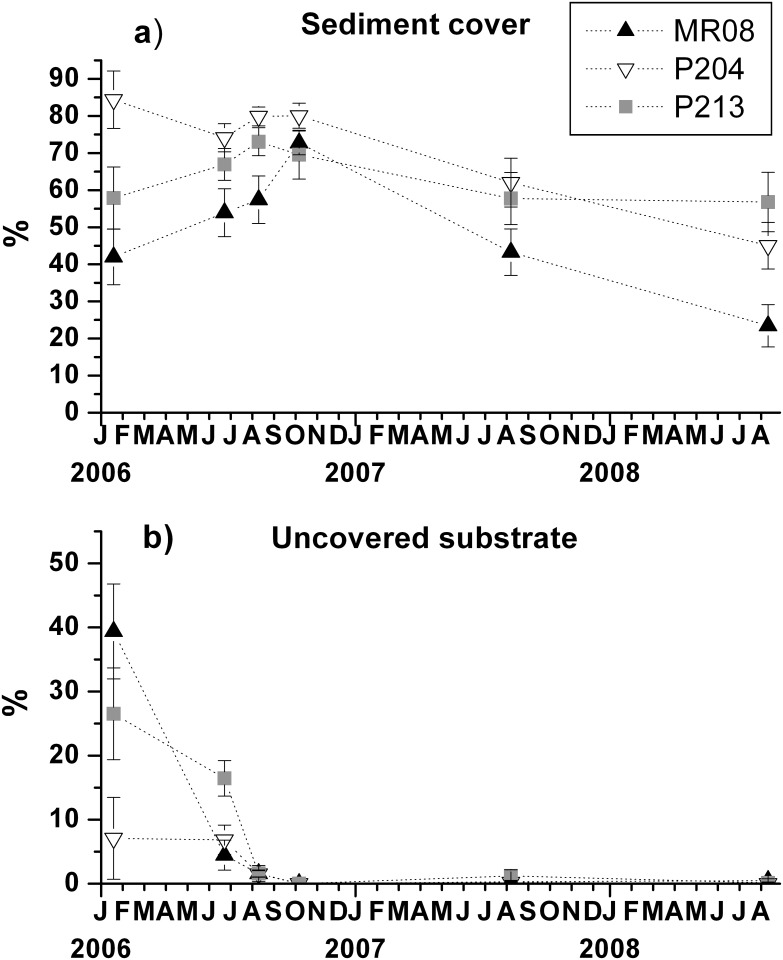
Temporal trends of occupation of space on experimental tiles. Temporal trends of (a) sediment percent cover and (b) proportion of substrate uncovered neither by sediments nor by organisms at each site (mean ± SE).

At the first sampling date (January 2006), five months after the deployment, tiles were largely covered by sediment, however several recruits were found. Sediment percent cover was very high at P204, the closest to the coast, reaching 84.4 ± 7.7% (Fig 5a), whilst the substrate uncovered neither by sediments nor by organisms was only 7.1 ± 6.4% (Fig 5b). The lowest sediment percent cover was observed at MR08 (42.0 ± 7.5%), the site farthest from the coast, with the highest proportion of uncovered substrate (39.4 ± 7.4%). The site P213 showed an intermediate situation. Patterns of sediment percent cover were in agreement with the sedimentation rates, which were nearly double at P204 (16 ± 7 cm yr^-1^) compared to P213 (6 ± 2 cm yr^-1^) and MR08 (8 ± 2 cm yr^-1^).

Overall, sediment cover remained very high until October 2006 afterwards it gradually declined, down to 56.8 ± 8.0% at P213, 45.0 ± 6.3% at P204, and 23.4 ± 5.7% at MR08 (Fig 5a). On the contrary, bare substrate decreased sharply until August 2006, being tiles progressively colonised by organisms that recruited in spring, and in October 2006 it was close to almost 0 (Fig 5b).

The assemblages on tiles appeared very heterogeneous since the beginning, showing a significant differentiation over the three-year study period both at local (plots) and at regional (sites) spatial scales (Table 4).

**Table 4:** PERMANOVA test on species assemblages on experimental tiles by Plots, Sites and Dates (Bray-Curtis similarities of square root-transformed data).

Source	df	SS	MS	Pseudo-*F*	*p* (perm)	Unique perms
Site	2	50777	25389	4.273	0.0002	9928
Date	5	51385	10277	8.613	0.0001	9937
Plot (Site)	6	20632	3439	2.882	0.0001	9905
Site × Date	10	28919	2892	2.424	0.0001	9862
Date × Plot (Site)	25	29829	1193	2.395	0.0001	9781
Res	147	73232	498			
Total	195	260920				

In January 2006, assemblages recruiting on travertine tiles included 7 taxa at MR08, 6 at P204, 10 at P213, belonging to 5, 4, and 6 classes respectively (Table 5). The most abundant taxa were serpulid polychaetes (6.5 ± 0.8%, mainly *Spirobranchus triqueter* (Linnaeus 1758)), juvenile encrusting calcareous Rhodophyta (5.8 ± 1.3%) and *Rocellaria dubia* (259 ± 72 ind. m^-2^) at MR08, turf of algae and hydroids (6.6 ± 1.2%) and *R*. *dubia* (230 ± 65 ind. m^-2^) at P204, and *Anomia ephippium* Linnaeus 1758 (4.5 ± 1.4%) and *R*. *dubia* (142 ± 44 ind. m^-2^) at P213. At the end of the study period, the assemblages were composed by 14 taxa at MR08, 12 at P204, 17 at P213, belonging to 7, 6, and 8, classes respectively (Table 5). At this stage, the most abundant taxa were *Lithophyllum incrustans* Philippi 1837 (22.8 ± 4.7%), *Lithothamnion minervae* Basso 1995 (19.3 ± 4.6%) and *Peyssonnelia polymorpha* (Zanardini) F. Schmitz 1879 (6.0 ± 1.1%) at MR08, turf of algae and hydroids (25.0 ± 5.9%), and *L*. *minervae* (4.7 ± 1.8%) at P204, and *L*. *minervae* (22.5 ± 10.2%) at P213. Although it was difficult to identify the species of sponges on the pictures, according to collected samples the most abundant sponges on tiles were *Tedania* (*Tedania*) *anhelans* (Vio in Olivi 1792), *Chondrosia reniformis* Nardo 1847, *Cliona viridis* (Schmidt, 1862), *Dysidea avara* (Schmidt 1862), *Phorbas fictitius* (Bowerbank 1866), *Dictyonella incisa* (Schmidt 1880), and *Aplysina* spp.

**Table 5:** Number of taxa belonging to the different classes found on experimental tiles for each site and date.

Main taxon	Site	January	June	August	October	August	August
		2006	2006	2006	2006	2007	2008
Turf of algae and hydroids	MR08	0	1	1	0	0	1
	P204	1	1	1	1	1	1
	P213	0	1	1	0	0	1
Florideophyceae	MR08	1	3	3	3	3	4
	P204	0	1	3	1	6	5
	P213	0	1	1	2	1	2
Demospongiae	MR08	1	1	2	2	5	3
	P204	0	1	1	1	0	3
	P213	2	2	4	1	6	9
Gymnolaemata	MR08	1	2	2	1	2	1
	P204	1	1	0	0	0	0
	P213	1	2	0	0	1	1
Ascidiacea	MR08	0	0	0	1	1	3
	P204	0	0	0	0	0	0
	P213	2	0	2	1	2	1
Bivalvia	MR08	2	1	1	1	1	1
	P204	2	1	1	1	1	1
	P213	2	2	2	1	1	1
Polychaeta	MR08	2	1	1	1	1	1
	P204	2	1	1	1	1	1
	P213	2	1	1	1	1	1
Anthozoa	MR08	0	0	0	0	0	0
	P204	0	0	0	0	0	1
	P213	1	0	0	0	0	1
**Total taxa**	MR08	7	9	10	9	13	14
	P204	6	6	7	5	9	12
	P213	10	9	11	6	12	17

**Fig 6:**
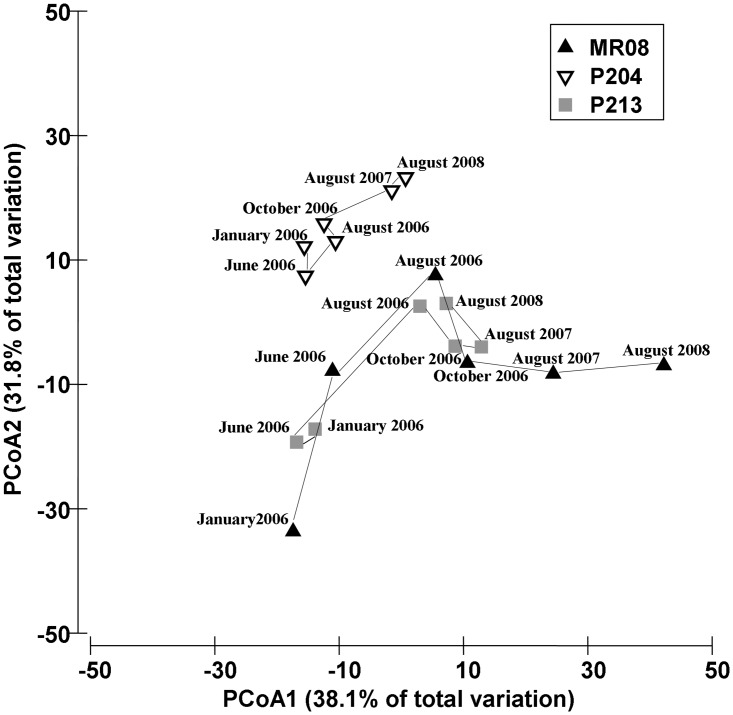
Differentiation of benthic assemblages on travertine tiles. PCoA ordination plot based on Bray-Curtis similarities of square root-transformed benthic assemblages data on travertine tiles: each point was the centroid of the observed similarity among Sites and Dates.

The PCoA ordination plot showed the similarity patterns of assemblages recruiting on travertine tiles (Fig 6). In the ordination plot, all assemblages shifted toward right over time, along the first PCoA axis, which explains 38.1% of total variation, while the second axis mainly accounts the difference among sites (31.8% of total variation). Assemblages at MR08 showed the major differentiation over time, especially during the first year when a shift from early-stage assemblages, dominated by fast-growing pioneer species (e.g., serpulid polychaetes), towards assemblages characterised by long-lived and structuring species (e.g., encrusting calcareous algae) occurred. Assemblages at P213 showed a shift between June 2006 and August 2006, when the early coloniser bivalve *A*. *ephippium* was partially replaced by encrusting calcareous algae and sponges. Assemblages at P204 appeared less variable compared to the other sites and were characterised by a remarkable amount of algal and hydroids mats (Fig 6).

**Fig 7:**
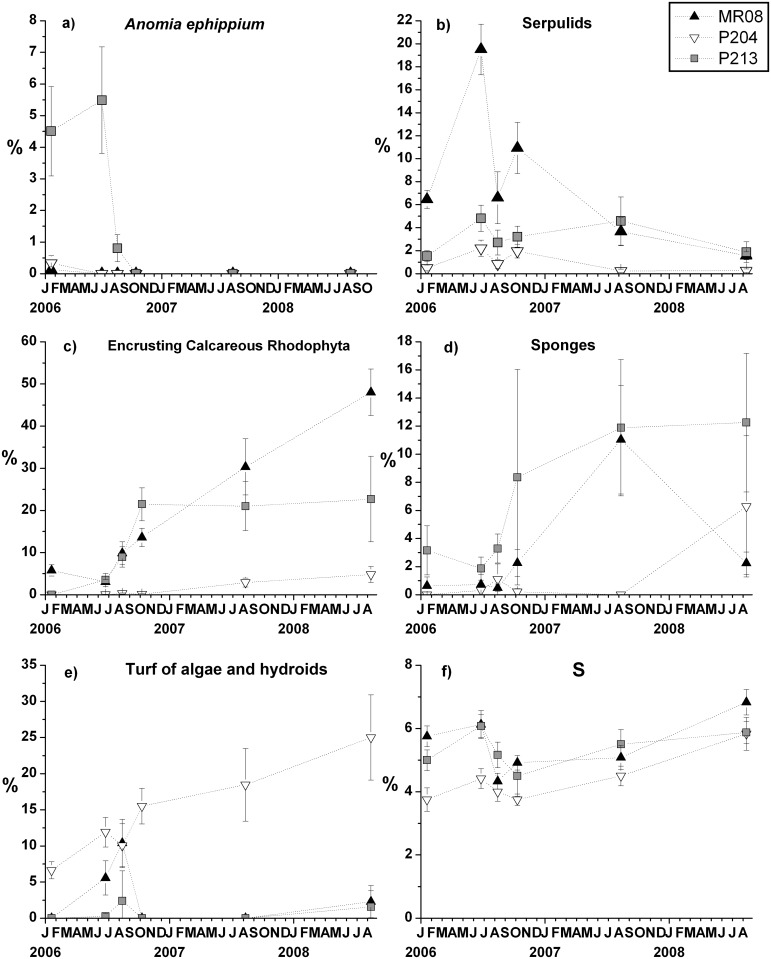
Temporal trends of percent cover. (a) *Anomia ephippium*, (b) serpulids, (c) encrusting calcareous Rhodophyta, (d) sponges, (e) turf of algae and hydroids and (f) species richness (*S*) at each site (mean ± SE).

The recruitment patterns of single taxa confirmed the general development trend of the assemblages. The early stage colonisers varied in time across plots or sites (Table 6). During the early recruitment phase, the most abundant species at P213 was *A*. *ephippium*, it increased up to 5.5 ± 1.7% until June 2006, and then it has been replaced by other species (Fig 7a). At MR08 the early stage colonisers were the serpulid polychaetes, mainly represented by *S*. *triqueter*. Serpulids, pooled together, reached 19.5 ± 2.2% cover at MR08 in June 2006 (Fig 7b), while their abundance was significantly lower in the other two sites (pair-wise tests: MR08 > P204 = P2013; *p*_*MC*_ < 0.01). After June 2006, serpulids showed a swinging trend and a final tendency to decrease at all sites.

**Table 6:** Summary of PERMANOVA test on the percent cover of selected taxa and species richness (*S*) at different Sites, among Plots within Sites and Dates.

	Site			Date			Plot (Site)			Site × Date			Date × Plot (Site)			Res
	MS	*F*_*2*,*6*.*03*_	*p*	MS	*F*_*5*,*25*_	*p*	MS	*F*_*6*,*147*_	*P*	MS	*F*_*10*,*25*_	*p*	MS	*F*_*25*,*147*_	*p*	MS
*Anomia ephippium*	55.07	6.85	0.0066 **	121.70	24.34	0.1573 ns	48.83	8.14	0.0002 ***	202.23	20.223	0.1877 ns	338.62	13.55	0.0001 ***	1.91
Serpulids	944.77	9.40	0.0422 *	239.38	9.84	0.0002 ***	101.97	10.14	0.0001 ***	111.13	4.5663	0.0012 **	24.34	2.42	0.0006 ***	10.06
Encrusting Calcareous Rhodophyta	5298.50	7.08	0.0447 *	2577.40	7.66	0.0003 ***	759.88	13.51	0.0001 ***	771.96	2.2932	0.0463 *	336.63	5.99	0.0001 ***	56.24
Sponges	448.97	4.90	0.0557 ns	275.81	3.45	0.0162 **	92.40	1.88	0.0838 ns	101.15	1.2662	0.2998 ns	79.89	1.62	0.0400 *	49.27
Turf of algae and hydroids	3380.00	5.54	0.0132 *	210.84	1.17	0.3512 ns	619.98	15.76	0.0001 ***	261.3	1.454	0.2232 ns	179.71	4.57	0.0001 ***	39.35
*S*	25.32	21.54	0.0121 *	12.69	8.80	0.0002 ***	1.17	0.82	0.5574 ns	2.1397	1.4837	0.2069 ns	1.44	1.01	0.4637 ns	1.43

Significant levels were indicated by the following symbols: ns = not significant,

* = *p* < 0.05;

** *p* < 0.01;

*** *p* < 0.001

At P213 and MR08 pioneer taxa were particularly abundant in the first year; afterwards they were replaced by late successional species. Since June 2006, the encrusting calcareous Rhodophyta, especially at MR08 (Fig 7c), and sponges, especially at P213 (Fig 7d), occupied more and more space, covering or replacing previous settlers. The recruitment of encrusting calcareous Rhodophyta on tiles started with patchy patterns, typical of planktonic propagules. In the meantime, colonial ascidians, massive and encrusting sponges growing on adjacent natural substrates colonised the tiles from the edges, by lateral encroachment.

A mixed turf of filamentous algae and hydroids was significantly more abundant on tiles deployed at P204 since the beginning of the experiment, and its cover slightly increased during the entire study period (Fig 7e).

The number of species (*S*) on the travertine tiles significantly varied among sites and dates in a consistent way (Table 6, Fig 7f). A significantly lower number of species was found at P204 compared to the other two sites during the entire study period (pair-wise tests: *p*_*MC*_ < 0.05). Between January and June 2006 species richness significantly increased at all sites (pair-wise tests: *p* < 0.05), then dropped until August 2006 (pair-wise tests: *p* < 0.05) and increased again between August 2007 and August 2008 (pair-wise tests: *p* < 0.05). At the end of the experiment (August 2008) mean *S* values were significantly higher than two years before (August 2006) (pair-wise tests: *p* < 0.01).

## Discussion

Mediterranean coralligenous habitats include a number of markedly different mesophotic biogenic temperate reefs that host very heterogeneous benthic assemblages. They are characterised by high spatial variability and limited temporal changes [41], unless anthropogenic disturbances [42] or natural anomalous events [43] occur. In the northern Adriatic Sea three typologies of coralligenous assemblages have been described. Although the observed differences among typologies of assemblages may be related to environmental conditions [21,24], several biological processes (e.g., larval settlement, post-larval recruitment, reproduction and growth modalities and intra- and interspecies space competition) could play a relevant role in the maintenance of species diversity at different spatial scales.

Although it is evident that natural materials cannot be exactly emulated by artificial ones, the latters have been proved to be effective in studying spatial and temporal variability of species recruitment [25,44–48], and effects of biotic and abiotic factors on recruitment processes [49–54]. At the end of the three-year recruitment experiment, assemblages found on experimental tiles differed from those on natural substrates. This might suggests that in the northern Adriatic Sea much longer time is needed for the development of the assemblages, as already highlighted in others study cases, for instance in the Ionian Sea [55], in the north Aegean Sea [56] and in the north-western Mediterranean Sea [41]. Indeed, the experiment was carried out to investigate the colonisation processes underlying the spatial heterogeneity of the benthic assemblages observed among outcrops, and after three years the most abundant taxa recorded on tiles were the same found on natural substrates, with consistent differentiation across sites. Ultimately, assemblages on travertine tiles at the end of the experiment represent a proxy of the differentiation among natural assemblages found between sites in terms of most abundant ecological groups.

**Fig 8:**
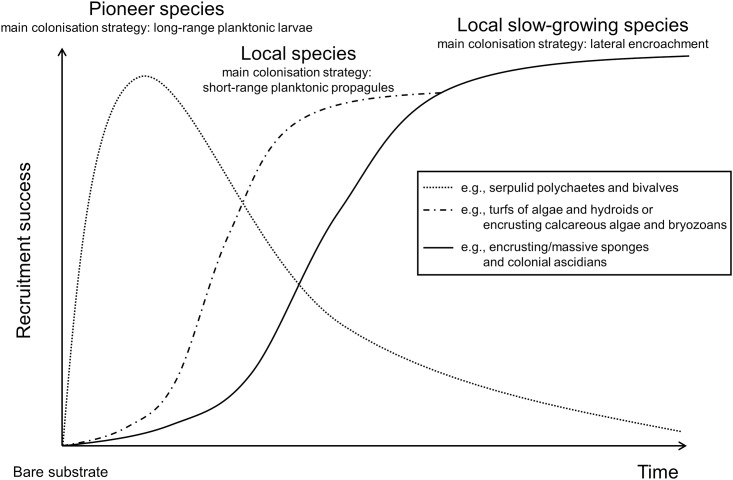
A conceptual model of recruitment processes on experimental tiles deployed on coralligenous outcrops of the northern Adriatic Sea. Species involved in the early colonisation stage largely depend on larvae availability and environmental conditions in the area. Over time colonisation by local species, living on natural substrates, become more relevant according to their life-cycle and dispersal strategies (see text for details).

The results of this experiment suggested a conceptual model for the dynamics of the processes structuring the assemblages (Fig 8). In the early-stage pioneer species, with high reproduction rate, high larval dispersion and fast growth, were the most abundant (e.g., serpulid polychaetes and the bivalve *Anomia ephippium*). Recruitment of sessile assemblages on tiles since the early-stages differed among plots (tens of meter apart) and sites (kilometres apart). These results suggest a high variability in larval supply at the investigated spatial scales, acting as a driver of the settlement processes of pioneer species. During the first 10 months of the experiment *Spirobranchus triqueter* was the most abundant species at the northern site MR08. *S*. *triqueter* has a very long reproductive period, with unpredictable peaks, more frequent from late summer to autumn [57,58]. Moreover, *S*. *triqueter* settlers have a gregarious behaviour, therefore the first recruiters may favour the fast growth of local populations [57]. The relatively low sedimentation rate at MR08, is among the environmental conditions that may have favoured settlement and recruitment of this serpulid polychaetes [59]. As many other pioneer species, *S*. *triqueter* is a poor competitor for space and it is often overgrown by other organisms [60], as observed after the first year, despite some recruitment occurred in autumn of the second year.

*Anomia ephippium* was the major coloniser of tiles at P213 till June 2006, later it was overwhelmed by other species. This species was rare in the surrounding natural assemblages [19] but its planktotrophic larva has a long dispersal ability, and several reproduction events occur during the year [61]. Therefore, dense populations of *A*. *ephippium*, occurring on man-made submerged structures in the northern Adriatic Sea [62,63] may have acted as the source of recruits. Larval settlement may have been facilitated by favourable local environmental conditions, including relatively low sedimentation rates [59].

The endolithic bivalve *Rocellaria dubia* is a common Mediterranean boring species, nevertheless its biology and ecology are poorly known. This species reproduces by planktonic larvae able to settle in crevices or holes in the substrate, and seems to be very tolerant to high sedimentation rates, thanks to its long siphons protected by aragonitic siphonal tubes [64,65]. Its ability to colonize the travertine substrates appeared very high at all study sites, where many specimens were found since the first sampling date, five months after the tiles deployment. The colonisation rate was higher than that observed on limestone tiles in shallow waters [66,67]. This highlights that *R*. *dubia* may play an important role by bioeroding the mesophotic coralligenous habitats of the northern Adriatic Sea.

Early colonisation at the site P204, the closest to the coast, was characterised by fast-growing turf of algae and hydroids. The high sedimentation rate measured at this site may have favoured the development of the turfs [68,69].

Encrusting calcareous algae and bryozoans showed a patchy colonisation in the early stages, possibly due to their ability to reproduce and disperse by free propagules, coming from the surrounding populations. In particular, *Lithophyllum incrustans* and many other coralline algae are characterised by a triphasic life cycle with an isomorphic alternation of generations. The populations of *L*. *incrustans*, studied in the British Isles, were largely made by asexual plants that reproduce by uninucleate apomeiotic bispores directly developing into asexual plants [70,71], providing an effective short-range dispersal.

Ten months after the deployment of tiles (June 2006), pioneer species were gradually replaced or overgrown by long-lived slow-growing species, which contribute to increase the species richness, complexity and structural features of the assemblages. In this phase, the colonisation of travertine tiles mainly occurred *via* lateral encroachment, especially by sponges and colonial ascidians (see examples in S1 Fig). Occupation of space differed among taxa. Sponges, thanks to their plasticity, tend to grow in the direction they encounter fewer obstacles and where conditions are more favourable. *Tedania* (*Tedania*) *anhelans* in the northern Adriatic Sea has been observed to grow from spring to summer, propagules develop on its surface, and reach their maximum size in July [72]. Besides asexual reproduction, this sponge releases larvae from May to October [72]. The sponge *Chondrosia reniformis* reproduces both sexually and asexually during all the year, the latter mainly occurs in spring and summer [72].

*Polycitor adriaticus* (Drasche 1883) and *Aplidium tabarquensis* Ramos-Espla 1991 were the most common colonial ascidians in the area and both belong to the order Aplousobranchia. Their colonies initially expand on tiles by lateral encroachment, later new colonies were observed. New colonies may originate asexually, by fission of pre-existing ones, or by settlement of larvae [73]. These colonial ascidians brood their larvae, resulting in the formation of dense population of closely related individuals [74–76].

Several researches highlighted the importance of native assemblages in determining the patterns of colonisation on artificial substrates [77–80]. In the present study, native assemblages represent a pool of potential colonisers that strongly influenced the colonisation dynamics at regional and local scale. In turn, geographical position and environmental conditions at each site were important in determining the assemblage structure at regional scale. Sedimentation is one of the major factors affecting the structure, biomass and metabolism of marine benthic communities [7]. Since the beginning, a thin layer of sediment covered most of the tiles at the site closest to the coast, thus hindering larval settlements and favouring turf forming species, which could proliferate under high sedimentation rates thanks to the effective vegetative propagation [68,69]. Instead, recruitment of encrusting calcareous Rhodophyta was higher at sites far from the coast, with lower rates of sediment deposition [59].

During the early colonisation stage the formation and differentiation of coralligenous assemblages on the northern Adriatic outcrops seemed to be driven by pioneer species characterised by long pelagic larval duration. Planktonic larvae of pioneer species may come from far away, according to the location of source populations, water circulation and seasonal biological cycles. Early experiments carried out by Relini et al. [81] on recruitment of pioneer species in Mediterranean coralligenous habitats revealed a seasonal pattern.

The colonisation processes observed on bare artificial substrates deployed directly on the natural substrates, are in substantial agreement with the space competition strategies of solitary and colonial species, based on ecological and life-history attributes [82]. Indeed, as observed in many subtidal rocky habitats worldwide, pioneer species were mainly represented by solitary organisms (e.g., serpulid polychaetes and bivalves), while colonial animals and crustose algae may outcompete them later, thank to indeterminate growth, larger independence from sexual reproduction, and lesser susceptibility to overgrowth. Resilience of the northern Adriatic coralligenous assemblages, and thus the maintenance of their diversity, appeared largely entrusted to asexual reproduction. At least, this seems to be the strategy adopted by the most abundant and structuring species in the area and at the investigated temporal scale.

Coralligenous outcrops in the northern Adriatic continental shelf have a very recent geological history. Considering the late Quaternary sea level changes and the current sedimentary processes [83,84], the formation of these outcrops started no more than 4–6,000 years ago. In recent decades, they have experienced the growing impact of trawling, able to dismantle entire portions of calcareous rocks, as well as dystrophic/anoxic crises and proliferation of mucilage that have led to mass mortality events of benthic species [85–87]. Exploring the mechanisms that underlie the formation and maintenance of the species diversity of these habitats is crucial both for improving our understanding of ecological processes and to implement appropriate conservation strategies.

## Supporting Information

S1 Fig Examples of colonisation succession on travertine tiles at each study site. (PDF)

S1 Dataset Complete dataset with percent cover of sessile organisms and number of siphonal tubes of *Rocellaria dubia* per tile, adjusted to the total readable area of each image. (XLSX)
