# Supplementary material for: Role of Recruitment Processes in Structuring Coralligenous Benthic Assemblages in the Northern Adriatic Continental Shelf
Source: PLoS One. 2016 Oct 4;11(10):e0163494. doi: 10.1371/journal.pone.0163494 (PMC5049771; doi:10.1371/journal.pone.0163494)
Supplement: S1 Fig — (PDF) [file pone.0163494.s001.pdf]

Structuring and differentiation of coralligenous benthic assemblages in the northern Adriatic continental shelf

Federica Fava, Marco Abbiati, Massimo Ponti

S1 - Supporting information

Examples of colonisation succession on travertine tiles at each site (MR08, P204, P213). In each column, photos show the same tile sorted by sampling date. The tile selected in P213 was not photographed in October 2006, due to poor visibility.

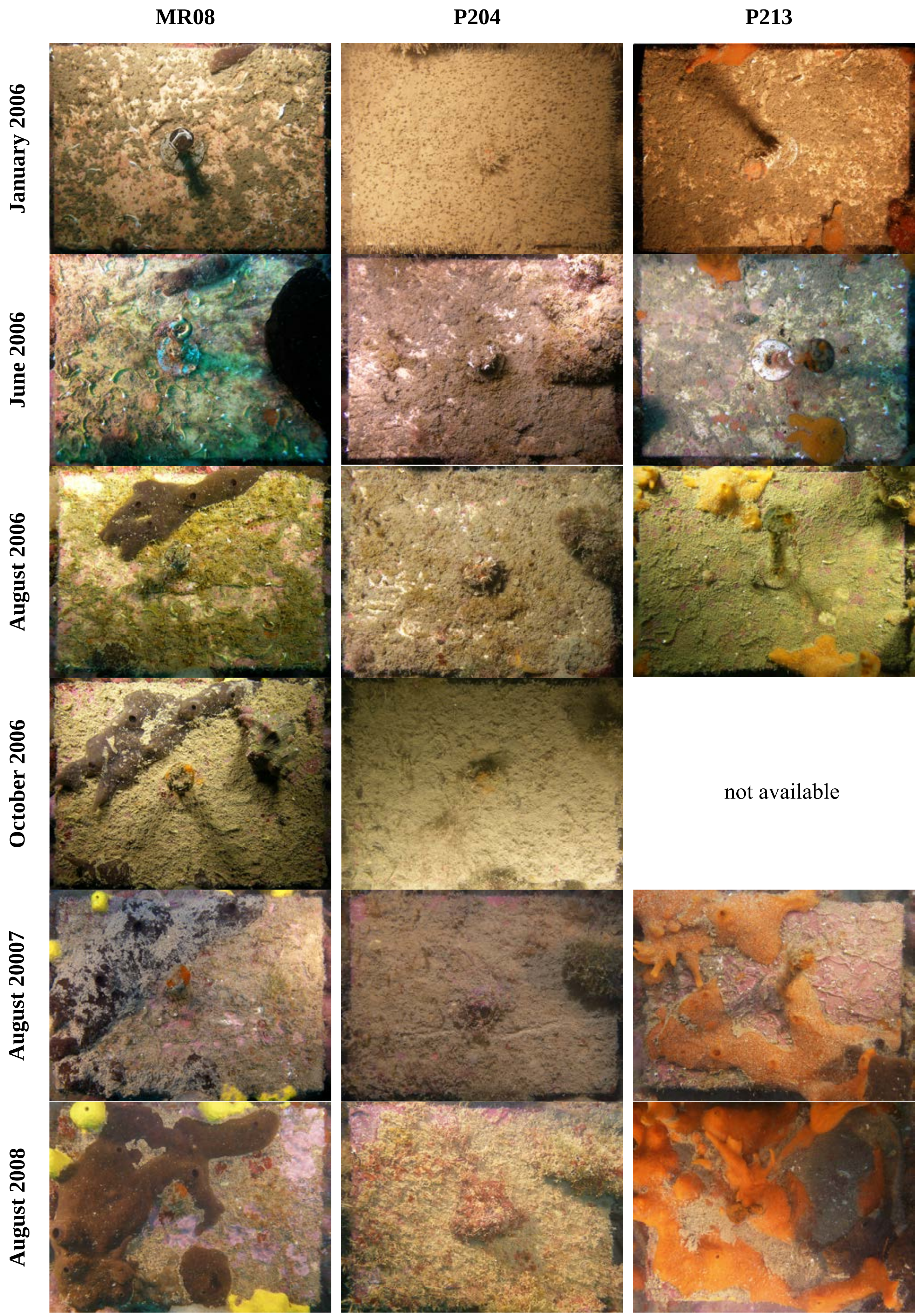
MR08
P204
P213
January 2006
June 2006
August 2006
October 2006
not available
August 20007
August 2008
